# Phone-based outpatients’ follow-up in mental health centers during the COVID-19 quarantine

**DOI:** 10.1177/0020764020979732

**Published:** 2022-02

**Authors:** Alessandro Gentile, Julio Torales, Marcelo O’Higgins, Pamela Figueredo, Joao Mauricio Castaldelli-Maia, Domenico De Berardis, Annamaria Petito, Antonello Bellomo, Antonio Ventriglio

**Affiliations:** 1 Department of Mental Health, ASREM, Termoli, Vasto, Italy; 2 Department of Psychiatry, School of Medical Sciences, National University of Asunción, Paraguay; 3 Department of Psychiatry, Medical School, University of São Paulo, Brazil; 4 Department of Mental Health, ASL 4 Teramo, Abruzzo, Italy; 5 Department of Clinical and Experimental Medicine, University of Foggia, Italy

**Keywords:** COVID-19, mental health, telepsychiatry, telemedicine, stress, pandemic, lockdown

## Abstract

**Background:**

The current COVID-19 pandemic is affecting mental health of global population and, particularly, of people suffering from preexisting mental disorders.

**Aims:**

This study aims to report on findings from a phone-based clinical follow-up conducted in two large catchment areas in Italy and Paraguay, during the COVID-19 lockdown, in order to provide psychiatric assessments and measure the level of stress related to the quarantine in a large sample of psychiatric outpatients.

**Methods:**

A clinical phone-based follow-up has been conducted in two large catchment areas in the province of Chieti (Vasto, Italy) and City of Asunción (Paraguay), during the COVID-19 national lockdown. The following rating scales have been employed: Hamilton Anxiety Rating Scale (HAM-A); Hamilton Depression Rating Scale (HAM-D); 18-items Brief Psychiatric Rating Scale (BPRS-18). The psychological distress related to the outbreak has been assessed employing the Impact of Event Scale – Revised (IES-R).

**Results:**

A total of 110 outpatients were consecutively included and followed among those reporting a stable phase of illness before the COVID-19 lockdown. Findings confirmed a significant increase of general psychopathology, anxiety and fear as well as mild levels of stress related to the quarantine. Also, significant weight gain during the lockdown was detected among patients.

**Conclusions:**

This study confirmed the impact of COVID-19 lockdown on mental health of people suffering from psychiatric disorders and may also add evidence on the employment of digital psychiatry in the current pandemic.

## Introduction

The current COVID-19 pandemic is affecting mental health of global population and has a significant impact on people suffering from preexisting mental disorders (Torales et al., 2020). Psychological distress related to the outbreak of pandemic and its impact on mentally ill patients has been described in the last months. Previously, during the SARS-CoV influenza in 2009, Mak et al. (2009) described long-term psychiatric morbidities among survivors with a cumulative incidence of psychiatric disorders (including post-traumatic stress disorders and depression) around 60% and an unexpected good adjustment among patients affected by severe psychotic disorders with autistic traits. Iasevoli et al. (2020) have recently shown that psychiatric patients reported higher level of stress, anxiety and depressive symptoms than the general population during the COVID-19 lockdown. Many other authors have described a worsening of psychopathological symptoms during the COVID-19 quarantine among those subjects affected by pre-existing mental disorders (Chevance et al., 2020; Cortese et al., 2020; Fernandez-Aranda, 2020; Garriga, 2020; Hao et al., 2020; Iancu et al., 2005; Kozloff et al., 2020; Narzisi et al., 2020; Yao et al., 2020; Wang et al., 2020). In addition, Muruganandam et al. (2020) reported that 30% of mentally ill patients has shown a clinically significant relapse during the quarantine.

Since the remote interventions and telepsychiatry have been employed worldwide to deliver assessments and clinical follow-up for psychiatric outpatients (Gentile et al., 2020; Liu et al., 2020; Torous et al., 2020), we describe the findings from our phone-based follow-up conducted during the COVID-19 lockdown in two large psychiatric catchment areas (in Italy and Paraguay): data on patients’ clinical outcomes and the impact of lockdown on their mental health will be discussed. Also, this report may add evidence to the efficacy of telemedicine and telepsychiatry in delivering remote assessments and interventions to mental health services users.

## Methods

A phone-based clinical follow-up and survey have been conducted from March 2020 to April 2020 for psychiatric outpatients based in a large area of Central-Southern Italy (Vasto, Province of Chieti) and Department of Psychiatry of University of Asunción, Paraguay. The survey has been also conducted in collaboration with the University of Foggia, Italy. All patients have been adequately informed about the anonymous use of clinical data collected for this observational study; consent was obtained by phone and registered in their clinical records (A.G. and J.T.). Data were treated with confidentiality, equality, and justice, respecting the Helsinki principles. Ethical approval has been obtained from the two Institutions involved (Polo Biomedico Adriatico, Vasto (C.H.), Italy, Department of Psychiatry of University of Asunción, Paraguay; A.G. and J.T.).

Patients, consecutively selected, were aged 18 to 65 years old, reporting a stable phase of illness in the last month (as documented in their clinical records: the definition of stable phase of illness was based on clinical assessments reported in the previous 6 months including no changes in the treatment protocols as well as the absence of suicidal ideation or attempts) before the COVID-19 lockdown in Italy (declared on 9th March 2020) and Paraguay (20th March 2020). Patients affected by substance abuse and major physical illness were excluded (in particular those medical conditions impacting on mental health, including neurological disorders, metabolic issues, systemic and oncologic diseases). Clinical follow-up has been conducted through phone calls (mostly) or video-calls during the COVID-19 lockdown. A comparison between patients’ clinical measurements and scores collected during the lockdown and their own previous clinical assessments, recorded before the COVID-19 pandemic, has been performed.

The assessment included clinical and socio-demographic data such as age, gender, education, employment, housing, marital status, medical history, mental illness years, current psycho-, and pharmacological therapy. Also clinical data before the lockdown have been retrospectively collected (including pre-lockdown clinical notes and ratings as extracted by the clinical records). Some rating scales have been administered by phone (through a phone-based clinical interview), including: Hamilton Anxiety Rating Scale (HAM-A: scoring anxiety as ‘absence’[<7], ‘mild’ [8–17], ‘moderate’ [18–24], ‘severe’ [>25]; Hamilton, 1959), Hamilton Depression Rating Scale (HAM-D: scoring depression as ‘mild’ [8–17], ‘moderate’ [18–24], ‘severe’ [⩾25]; Hamilton, 1967). General psychopathology has been scored with 18-items Brief Psychiatric Rating Scale (BPRS; Overall & Gorham, 1962). The psychological distress related to the outbreak has been assessed employing the Impact of Event Scale – Revised (IES-R; Weiss & Marmar, 1996). IES-R is a 22-item self-report, validated in Italian and Spanish (for Paraguay), of distress caused by a traumatic event: total scoring ranges from normal (0–23), mild (24–32), moderate (33–36) stress with a cut-off score of 24 used to detect a post-traumatic stress disorder. In addition, prevalent emotions among Serenity, Optimism, Fear, Hope – Hopelessness, Pessimism, Anger, were surveyed.

Statistical analyses performed with Statview (SAS Corp., Cary, NC.) included means ± standard deviations (SD) or percentages (%). Continuous data were compared by analysis of variance (ANOVA) methods (*F*), and categorical data by contingency tables (χ^2^). Findings are considered statistically significant with a two-tailed value less than 0.05.

## Results

110 patients were recruited, 60 based in the Province of Chieti (City of Vasto), Italy, and 50 from Asunción, Paraguay, 60 females and 50 males. Patients’ mean age was 38.6 ± 14.1 with 13.3 ± 3.77 years of education and 46.3% rate of employment (*n* = 51/110). Of them, 32.7% were married (*n* = 36) and 69.1% living in family (*n* = 76). Psychiatric diagnoses were: major depression (*n* = 37, 33.6%) > psychosis (*n* = 28; 25.4%) > bipolar disorder (*n* = 18; 16.3%) > anxiety disorders (*n* = 15; 13.6%) > personality disorders (*n* = 12; 10.9%). All diagnoses have been confirmed by expert clinicians (AG, JT) according to the Diagnostic and Statistical Manual of Mental Disorders [diagnoses among outpatients were based on a DSM-5-based structured clinical interview at first consultation and underpinned by the clinical follow-up (DSM*–*5; APA], 2013)]. Patients’ years of mental illness were 8.26 ± 8.79 with 0.72 ± 1.11 lifetime hospitalizations for acute psychopathological episodes: we recruited patients reporting a stable phase of illness before the COVID-19 lockdown. Also, 21.8% of patients were affected by a non severe physical comorbidity since we excluded major physical problems potentially influencing patients’ subjective wellbeing. None reported hospitalizations during the national quarantine, 17.2% (*n* = 19) reported current suicidal ideation. Ongoing treatments included: antidepressants (*n* = 51; 46.3%) > antipsychotics (*n* = 32; 29.1%) > antipsychotics + mood stabilizers (*n* = 22; 20.0%) > psychotherapy (*n* = 5; 4.54%). 32.7% (*n* = 36) of followed patients required changes of the ongoing treatments during the quarantine and 4.54% of patients continued their own psychotherapy sessions online. 62 patients (56.3%) reported lifestyle changes during the quarantine including: 32.7% variations in their eating pattern > 4.54 % variations of sleeping pattern > 2.72% increased alcohol consumption > others (16.3% including more reading and gaming). Also, 7.27% patients (*n* = 8) reported weight gain during the lockdown.

Self-reported prevalent emotions from the patients ranked: Fear 24.5% (27) > Optimism 20% (*n* = 22) > Pessimism 14.5% (*n* = 16) > Hope 13.6% (*n* = 15) > Hopelessness 10.9% (*n* = 12) > Serenity 9.1% (*n* = 10) > Anger 7.27% (*n* = 8). Patients’ total mean score reported at the Impact of Event Scale – Revised scale was 29.8 ± 15.5, documenting a ‘mild’ impact of global major event (COVID-19 lockdown) on their subjective stress levels. In addition, psychopathology ratings scored at BPRS 13.1 ± 6.45, HAM-A 16.6 ± 9.47, HAM-D 11.4 ± 7.26, within 30 days before the lockdown, whereas scored BPRS 14.8 ± 8.26, HAM-A 18.5 ± 9.68, HAM-D 11.9 ± 7.56, during the lockdown. Changes in total scores were BPRS +1.70 (*p*=0.0002), HAM-A +1.90 (*p* = .0025), HAM-D +0.50 (*p* = .326), respectively, describing a significant increase in general psychopathology and anxiety symptoms.

Bivariate analyses described significantly associated factors with the increasing patients’ stress related to COVID-19 lockdown (Table 1): increased levels of general psychopathology (+ 1.70 at BPRS total score; *p* = .0004), increased levels of anxiety (+1.90 at HAM-A; *p* = .006), need of changing the ongoing psychiatric treatments (*p* = .0010), weight gain during the quarantine (*p* = .0036), prevailing emotions of fear (*p* = .0002).

**Table 1.** Associated factors to mentally ill outpatients’ stress related (impact of event scale – revised, IES-R) to COVID-19 lockdown (*N* = 110).

Factors	*M* ± *SD* or percentage (%, *n/N*)	*p*-Value (*F or* χ^2^)
Current age (years old)	38.6 ± 14.1	.196 (1.91)
Sex (female)	54.5 (60)	.614 (0.49)
Education (years)	13.3 ± 3.77	.233 (1.43)
Employment (yes)	46.3 (51)	.851 (0.03)
Marital status (married)	32.7 (36)	.135 (2.25)
Housing (own family)	69.1 (76)	.921 (0.08)
Years of illness	8.26 ± 8.79	.315 (1.01)
Diagnosis		
Depression	33.6 (37)	.060 (2.09)
Bipolar disorder	16.3 (18)	
Psychosis	25.4 (28)	
Personality disorder	10.9 (12)	
Anxiety	13.6 (15)	
Hospitalizations		
Previous	0.72 ± 1.11	.156 (2.03)
During the COVID-19	0.00 ± 0.00	
Treatments		
Antidepressants	46.3 (51)	.413 (0.96)
Antipsychotics	29.1 (32)	
Antipsychotics + Mood stabilizers	20.0 (22)	
Psychotherapy	4.54 (5)	
Treatment changes during the COVID-19 (*yes*)	32.7 (36)	**.0010** (11.3)
Suicidal ideation during the COVID-19 (yes)	17.2 (19)	.527 (0.40)
Lifestyle changes during the COVID-19 (yes)	56.3 (62)	.162 (1.51)
Physical problems during the COVID-19 (weight gain)	7.27 (8)	**.0036** (8.83)
Prevalent emotions		
Hope	13.6% (15)	**.0002** (4.48)
Hopelessness	10.9% (12)	
Fear	24.5% (27)	
Anger	7.27% (8)	
Serenity	9.1% (10)	
Optimism	20% (22)	
Pessimism	14.5% (16)	
IES-R score	29.8 ± 15.5	–
BPRS		
Before the lockdown	13.1 ± 6.45	**.0004** (13.3)
During the lockdown	14.8 ± 8.26	
Changes (during – before the COVID-19)	+1.70	
HAM-A		
Before the lockdown	16.6 ± 9.47	**.006** (7.74)
During the lockdown	18.5 ± 9.68	
Changes (during – before the COVID-19)	+1.90	
HAM-D		
Before the lockdown	11.4 ± 7.26	.093 (2.86)
During the lockdown	11.9 ± 7.56	
Changes (during – before the COVID-19)	+0.50	

*Note.* SD = standard deviation; COVID-19 = Coronavirus disease-19; IES-R = Impact of Event Scale – Revised; BPRS = Brief Psychiatric Rating Scale; HAM-A = Hamilton Anxiety Rating Scale; HAM-D = Hamilton Depression Rating Scale.

Bold entries are used for statistically significant values.

## Discussion

This study described findings from a phone-based follow-up conducted in two large catchment areas in the province of Chieti (Vasto, Italy) and City of Asunción (Paraguay), during the COVID-19 lockdown, in order to provide psychiatric assessments and proper interventions for psychiatric outpatients, even if remotely. In addition, levels of stress related to the quarantine have been measured employing the IES-R scale along with the routine psychopathology ratings.

Our results confirmed an expected impact of COVID-19 quarantine on mental health of subjects affected by preexisting mental disorders (Chevance et al. 2020; Cortese et al. 2020; Fernandez-Aranda, 2020; Torales et al., 2020). The level of stress scored as ‘*mild*’ at IES-R: this may reflect that most of the assessments were performed in the mid March and the beginning of April 2020, and tested the short-mid term psychological reaction to the lockdown. Also, the rating employed is aimed to measure those levels of stress tightly related to the stressful/meaningful event. A significant increase in general psychopathology (BPRS) and anxiety (HAM-A) were also found, as expected, as well as a prevalent emotion of fear: most of studies conducted in the general population reported this trend of anxious reaction and fear, globally (Garriga, 2020; Hao et al., 2020; Iancu et al., 2005; Kozloff et al., 2020; Narzisi et al., 2020; Yao et al., 2020; Wang et al., 2020). The increase of depressive symptoms was not significant probably due to the short-mid term assessment: this may suggest that depressive reactions may be mostly expected in the mid-long term (Iasevoli et al., 2020; Torales et al., 2020). The need of changing some previous ongoing psychiatric treatments also may suggest the relevance of changes in psychopathology and anxiety, as described. In addition, as globally reported, weight gain was a significant physical issue related to stress, also due to sedentary lifestyle and lack of physical activity (Torales et al., 2020): this result may need further research since changes in lifestyle, including more eating, were reported in 56.3% participants but were poorly associated to IES-R score.

These findings report on a real-world investigation conducted in two large clinical outpatient settings. They may confirm the usefulness of tele-psychiatry in order to provide psychiatric outpatients with a proper assessment and follow-up, even if remotely, and may suggest future protocols of interventions in the COVID-19 era. Consequently, psychiatric services should be technologically supported and similar digital experiences should be promoted in order to safeguard patients as well as mental health care professionals.

Limitations may include a small sample, lack of a mid-long term follow-up and reliability of data, since they were collected by phone or videocalls. Also, we did not compare data collected in Italy *versus* Paraguay since we did not focus on variables related to nationality or socio-cultural aspects. Moreover, lockdown has been a global experience with a similar social impact through the countries. Differences among diagnostic classes were not investigated since levels of stress was not significantly associated to diagnosis (Table 1) and numbers were small.

## Conclusion

This study confirmed the impact of COVID-19 lockdown on mental health of people suffering from psychiatric disorders and may also add evidence on the employment of digital psychiatry and its sensitivity in detecting mental health issues in order to deliver proper interventions during the current pandemic.
